# Prevalence, incidence and mortality of hypertrophic cardiomyopathy based on a population cohort of 21.9 million in China

**DOI:** 10.1038/s41598-022-20042-9

**Published:** 2022-11-05

**Authors:** Ying Bai, Jian-Peng Zheng, Feng Lu, Xi-Lin Zhang, Chang-Ping Sun, Wei-Hua Guo, Yi-Xi Zou, Gregory Y. H. Lip, Xu-Bo Shi

**Affiliations:** 1 Cardiovascular Center, Beijing Tongren Hospital, Capital Medical University, Beijing, China; 2 Beijing Municipal Health Commission Information Center, Beijing, China; 3 Tianjin Key Laboratory for Control Theory and Application in Complicated Systems, Tianjin University of Technology, Tianjin, 300384 China; 4 School of Electrical Engineering and Automation, Tianjin University of Technology, Tianjin, 300384 China; 5 Cardiovascular Department, Dongzhimen Hospital, Beijing University of Chinese Medicine, Beijing, 101121 China; 6 Department of Cardiac Surgery, Beijing Anzhen Hospital, Capital Medical University, Beijing, 100029 China; 7 Liverpool Centre for Cardiovascular Science, University of Liverpool and Liverpool Heart and Chest Hospital, Liverpool, UK; 8 Aalborg Thrombosis Research Unit, Department of Clinical Medicine, Aalborg University, Aalborg, Denmark

**Keywords:** Cardiac hypertrophy, Mechanisms of disease

## Abstract

There are limited studies on the prevalence and incidence of clinically diagnosed hypertrophic myocardiopathy (HCM) and its mortality in the Chinese population, and the projected population burden of HCM over the next decades. We collected data on HCM and its mortality from the Beijing Municipal Health Commission Information Center (BMHCIC) database and estimated the prevalence and incidence based on the whole Beijing population. Calculation of population trends was performed using annual percentage change (APC) and average annual percentage change (AAPC). Finally, future HCM incidence was built by modelling projection of HCM to the next decades using Poisson regression analysis and Gray Model 1,1(GM [1,1]). The prevalence of HCM was 0.0069% (95%CI, 0.0065–0.0072%; *N* = 1343) in 2010, rising to 0.076% (95% CI, 0.074–0.077%; *N* = 16,616) in 2019, and the incidence of HCM was 6.85 per 100 000 person-year in 2010, rising to 11.76 per 100 000 person-year in 2019. Males had higher prevalence and incidence of HCM than females. The APPC for the rising incidence of HCM was 5.8% and the expected numbers will double increase in 2029 by assuming the same increase trend as the last decades. HCM had increased annual incidence of HF (APPC: 8.4, 4.4–12.6, *p* < 0.05), and relatively stable annual incidence of mortality (APPC: 1.2%, − 2.3% to 4.8%, *p* > 0.05) during the studied period. Males had lower mortality (2.70% vs. 4.20%, *p* < 0.001) than females. The calculated HCM prevalence was much lower compared to prior screening studies from 2004, although the predicted HCM incidence would double over the next decades. HCM was associated with a stable risk of mortality during the studied period.

## Introduction

The incidence of hypertrophic myocardiopathy (HCM) varies widely around the world, ranging from 0.02 to 0.2% either in the western countries^1–3^ or in Asian countries^4–7^. HCM was considered as a genetic targeted disease developing either in childhood or in adulthood^8,9^, but was less identified unless symptoms occurred^10,11^. Therefore, the patients had less recognition of HCM before its symptoms and HCM was usually diagnosed when complications develop, such as heart failure (HF)^12^, stroke^13^, atrial fibrillation (AF)^14^ or sudden cardiac death.

Knowledge of the prevalence of HCM is important for exploring its impact on its associated complications, morbidity and mortality^10,15,16^. An increased prevalence and incidence of HCM in Asian countries in recent decades is probably due to methodological development in the echocardiographic interpretation of HCM and increased clinical awareness or recognition^6^. However, the prevalence has only been reported in a small numbers and limited age ranges^7^.

There are few studies focusing on the change in HCM incidence in the Chinese population. In this registry study of 21.90 million Chinese population, we investigated the incidence of HCM in all age groups (0 to more than 100 years old) in China from 2010 to 2019. Second, we estimated the development of this disease with the estimation of the trends using annual percentage change (APC) and average annual percentage change (AAPC). Third, we explored the temporal incidence of HCM-related complications such as HF, AF, stroke and mortality.

## Materials and methods

The current Beijing District consists of 8 urban areas and 8 suburban areas. The inhabitants ranged from 19.62 million in 2010 to 21.90 million in 2019 according to government reports (http://tjj.beijing.gov.cn).

### Source of database

This whole Beijing population-based registry study used the Beijing Municipal Health Commission Information Center (BMHCIC) database. BMHCIC is a mandatory health surveillance and supervision government agency requiring the medical information uploaded from all the 153 hospitals/centers located in the overall Beijing area. The registry covered the demographics information including sex, age, ethnicity, registered date, registered center, contact information, variation of diseases and vital status during each hospitalization. The study protocol conformed to the ethical guidelines of the Declaration of Helsinki and was approved by the Ethical committee of Beijing Tongren Hospital, Capital Medical University. Informed consent was waived due to anonymized and unidentified information for the analysis.

### Diagnosis of HCM cases and death

HCM was diagnosed according to the recommendations of American Heart Association (AHA)^17^. Patients with simple left ventricular hypertrophy (LVH), which did not conform to the HCM diagnosis criteria were excluded. The diagnosis was collected at each registry using the claims for diagnostic codes *(International Classification of Diseases, Tenth Revision, Clinical Modification; ICD-10-CM)* with I42.101 for obstructive HCM, I42.201 for unobstructive HCM, I42.202 for HCM (which was defined as undefined HCM in this study), and I42.203 for apical HCM. The diagnosis was established by cardiologists and sonographers for HCM (the clinical diagnostic data used in this study are shown in Supplementary Table I), and then the accuracy was further validated by the experts invited by BMHCIC after being uploaded. The first time of diagnosis with HCM was considered as the date of onset for the condition.

### Statistics analysis

The prevalence of HCM was calculated using the cumulative number of survived HCM cases divided by the number of total populations in that referred year. The annual incidence of HCM was calculated with the number of newly diagnosed HCM cases divided by the population in that referred year and indicated as per 100,000 person-year. Mortality, rate of HF and rate of Implantable Cardioverter-defibrillator (ICD) implantation were calculated by dividing the events using the corresponding HCM number in that referred year. Comparisons of categorical variables were calculated using a chi-square test, while their change with year were calculated with Linear-by-Linear Association. The occurrence of incident HCM cases with year was assumed to conform to Poisson distribution and the time trends was evaluated with Joinpoint 4.9.0.0 (https://surveillance.cancer.gov/joinpoint/) with APC for each segment and AAPC for the whole trends^18^. Incidence rate ratio (IRR) and 95% confidence interval (CI) of HCM cases with the potential effect of increased age and sex was calculated with the assumption of conforming to Poisson distribution. Poisson regression models and GM (1,1) were used to predict incident HCM cases in the next decades^19^. Annual prevalence and incidence of subtypes of HCM (i.e. Apical HCM, obstructive HCM, unobstructive HCM and undefined HCM) were also calculated. SPSS, Matlab and Joinpoint software 4.9.0.0 were used for the calculation of the study. *P* value < 0.05 was considered statistically significant.

### Ethical statement

The study protocol conformed to the ethical guidelines of the Declaration of Helsinki and was approved by the Ethical committee of Beijing Tongren Hospital, Capital Medical University. Informed consent was waived due to anonymized and unidentified information for the analysis.

## Results

**Figure 1:**
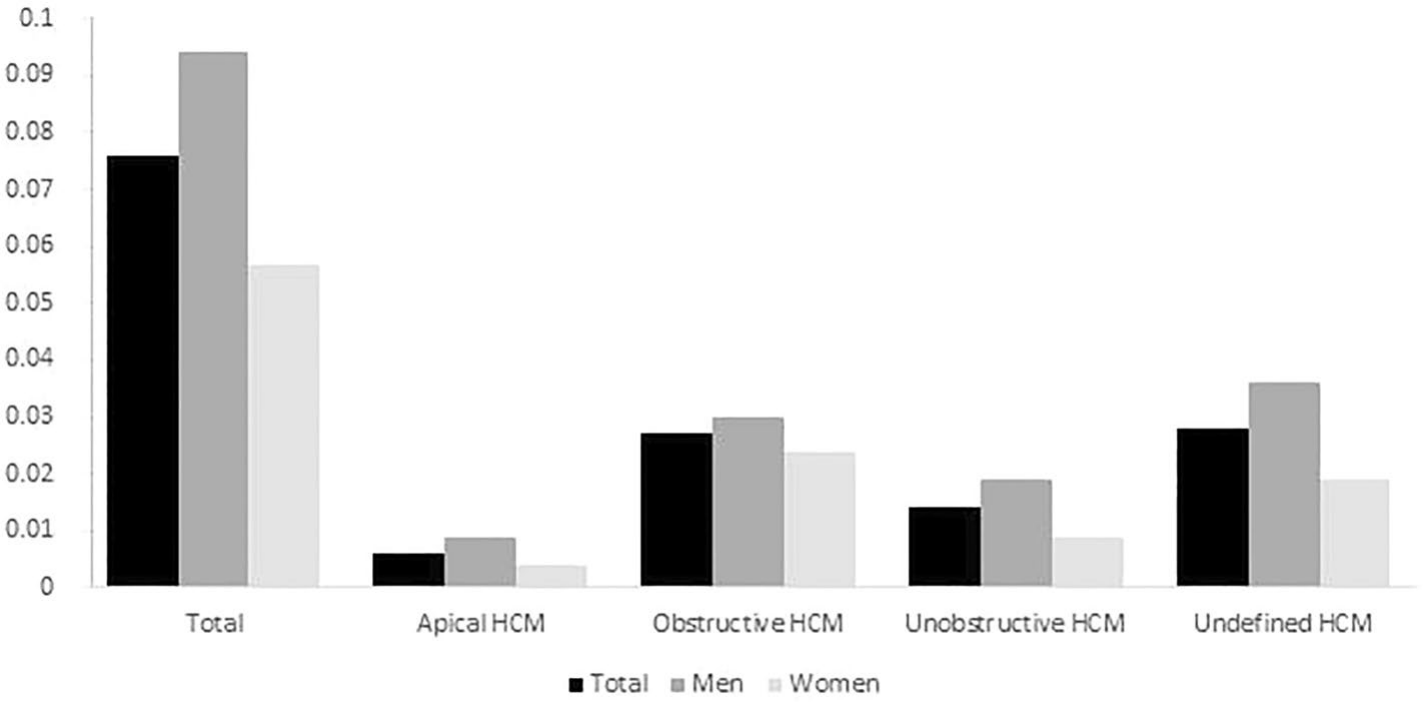
Prevalence of hypertrophic cardiomyopathy and its subtypes in 2019 in the total, males and females. HCM, hypertrophic myocardiopathy.

In 2010, the overall prevalence of survived HCM survival was 0.0069% (95%CI, 0.0065–0.0072%; *N* = 1343) with 0.0081% (95CI%, 0.0076–0.0087%; *N* = 824) in males and 0.0055% (95%CI, 0.0050–0.0059%, *N* = 519) in females (Supplementary Table II). In 2019, the overall prevalence of survived HCM was up to 0.076% (95% CI, 0.074–0.077%; *N* = 16,616) with 0.094% (95% CI, 0.092–0.096%, *N* = 10,537) in the male, 0.057% (95% CI, 0.055–0.058%, *N* = 6079) in the female population and the ratio of males to females of 1.65:1 (Fig. 1) The prevalence of subtypes of HCM are shown in Fig. 1 as well.

**Figure 2:**
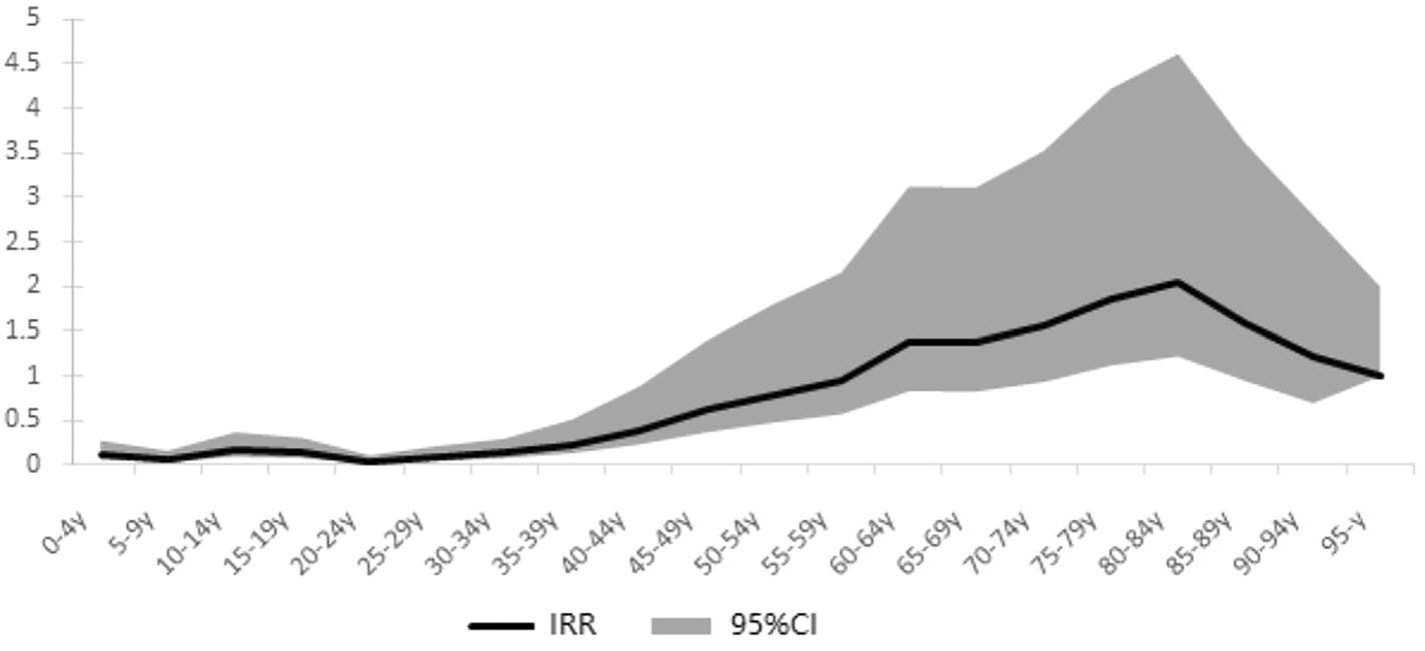
Incidence (95% CI) of hypertrophic cardiomyopathy with Ageing. HCM, hypertrophic myocardiopathy.

From 2010 to 2019, a total of 17,085 newly diagnosed HCM were identified from the current registry, with an incidence increasing from 6.85 (6.48–7.21) in 2010 to 11.76 (11.30–12.21) in 2019 per 100 000 person-year (Table 1). Of these, the incidence of HCM increased with age overall, and in the male and female population. The incidence of HCM increased with age with the peak incidence in the age range of 80 to 84 years after adjustment of sex using Poisson distribution analysis as shown in Fig. 2. The age-specific incidence also increased with age in males (Supplementary Fig. IA) and females (Supplementary Fig. IB). The IRR of males to females was 1.73 (95% CI: 1.68–1.79) based on Poisson distribution analysis after adjustment of age.

**Table 1:** Incidence of hypertrophic cardiomyopathy.

	Newly diagnosed HCM cases	Population	Incidence (95% CI) Per 100 000 person-years
**Total**			
2010	1343	19,612,368	6.85 (6.48, 7.21)
2011	1320	20,231,159	6.52 (6.17, 6.88)
2012	1368	20,767,977	6.59 (6.24, 6.94)
2013	1344	21,246,815	6.33 (5.99, 6.66)
2014	1479	21,703,661	6.81 (6.47, 7.16)
2015	1659	21,875,603	7.58 (7.22, 7.95)
2016	1829	21,957,104	8.33 (7.95, 8.71)
2017	1989	21,947,103	9.06 (8.66, 9.46)
2018	2179	21,920,099	9.94 (9.52, 10.36)
2019	2575	21,904,097	11.76 (11.3, 12.21)
**Males**			
2010	824	10,126,430	8.14 (7.58, 8.69)
2011	841	10,318,297	8.15 (7.60, 8.70)
2012	875	10,733,216	8.15 (7.61, 8.69)
2013	833	10,975,131	7.59 (7.07, 8.11)
2014	912	11,184,057	8.15 (7.63, 8.68)
2015	994	11,258,031	8.83 (8.28, 9.38)
2016	1203	11,262,398	10.68 (10.08, 11.29)
2017	1273	11,240,396	11.33 (10.7, 11.95)
2018	1396	11,223,394	12.44 (11.79, 13.09)
2019	1629	11,206,391	14.54 (13.83, 15.24)
**Females**			
2010	519	9,483,938	5.47 (5.00, 5.94)
2011	479	9,912,861	4.83 (4.40, 5.26)
2012	493	10,034,761	4.91 (4.48, 5.35)
2013	511	10,271,684	4.97 (4.54, 5.41)
2014	567	10,519,604	5.39 (4.95, 5.83)
2015	665	10,617,573	6.26 (5.79, 6.74)
2016	626	10,694,705	5.85 (5.39, 6.31)
2017	716	10,706,706	6.69 (6.20, 7.18)
2018	783	10,696,705	7.32 (6.81, 7.83)
2019	946	10,697,705	8.84 (8.28, 9.41)

*HCM* hypertrophic myocardiopathy.

The annual incidence of subtypes of HCM is shown in Supplementary Table III. This showed newly diagnosed apical HCM rising from 0.46(0.36,0.55) to 1.17(1.03,1.31), obstructive HCM from 2.22(2.01,2.43) to 4.62(4.34,4.91) and unobstructive HCM from 1.25(1.09,1.41) to 1.95(1.77,2.14) and undefined HCM from 2.92(2.68,3.16) to 4.01(3.74,4.27).

Incidence rate ratio of subtypes of HCM with ageing was shown in Supplementary Fig. II, which showed the higher IRR in the relatively elder age range.

The IRR of males to females had a range of 1.29:1 to 2.40:1 after the adjustment of age according to Poisson distribution analysis (Supplementary Table IV).

### Temporal trends from 2010 to 2019

As shown in Table 2, the increasing trends were seen in overall (AAPC = 5.8%, *p* < 0.05), and amongst males (AAPC = 6.6%, *p* < 0.05) and females (AAPC = 5.3%, *p* < 0.05) from 2010 to 2019. The APC slightly decreased since 2010 to 2013 and sharply increased from 2013 to 2019 in overall and amongst males and females (all *p* < 0.05). The AAPC for the total population increased to 7.0 and the AAPC increased in males to 7.4 and females to 6.3 (All *p* < 0.05).

**Table 2:** Temporal trends of incidence of hypertrophic cardiomyopathy.

	Year Range	Joinpoint	APC (95% CI)	*p* value	AAPC (95% CI)	*p* value
**Hypertrophic cardiomyopathy**						
Total	2010–2013	1	− 3.2 (− 8.5–2.5)	> 0.05		
	2013–2019	1	10.5 (8.8–12.3)	< 0.05		
	2010–2019	1			5.8 (4.0–7.5)	*p* < 0.05
	2010–2019	0			7.0 (4.5–9.4)	*p* < 0.05
Males	2010–2014	1	− 0.3 (− 4.7–4.3)	> 0.05		
	2014–2019	1	12.5 (9.6–15.4)	< 0.05		
	2010–2019	1			6.6 (4.6–8.6)	*p* < 0.05
	2010–2019	0			7.4 (4.7–10.1)	*p* < 0.05
Females	2010–2013	1	− 2.7 (− 15.6–12.1)	> 0.05		
	2013–2019	1	9.5 (5.2–14.0)	< 0.05		
	2010–2019	1			5.3 (1.0–9.7)	*p* < 0.05
	2010–2019	0			6.3(3.7–8.9)	*p* < 0.05
0–9 years old	2010–2019	0			− 4.8(− 1.3–11.3)	*p* > 0.05
10–29 years old	2010–2019	0			25.3(20.9–29.9)	*p* < 0.05
30–59 years old	2010–2019	0			6.9(5.2–8.7)	*p* < 0.05
60 + years old	2010–2019	0			− 1.6(− 4.7–1.6)	*p* > 0.05
**Death in hypertrophic cardiomyopathy**						
Total	2010–2019	0			1.2 (− 2.3–4.8)	> 0.05
Males	2010–2019	0			1.5(− 2.5–5.7)	> 0.05
Females	2010–2019	0			0.4(− 6.0–7.2)	> 0.05

*HCM* hypertrophic myocardiopathy, *APC* Annual percentage change, *AAPC* average annual percentage change.

We calculated temporal trends by categorized age of 0–9 years, 10–29 years, 30–59 years and $\ge$ 60 years and found the AAPC showed increasing trends in the age range of 10–59 years. The increasing trends of subtypes of HCM are shown in Supplementary Table V, which showed increasing trends in all the subtypes of HCM of overall.

### Prediction of new cases of HCM beyond 2019

**Figure 3:**
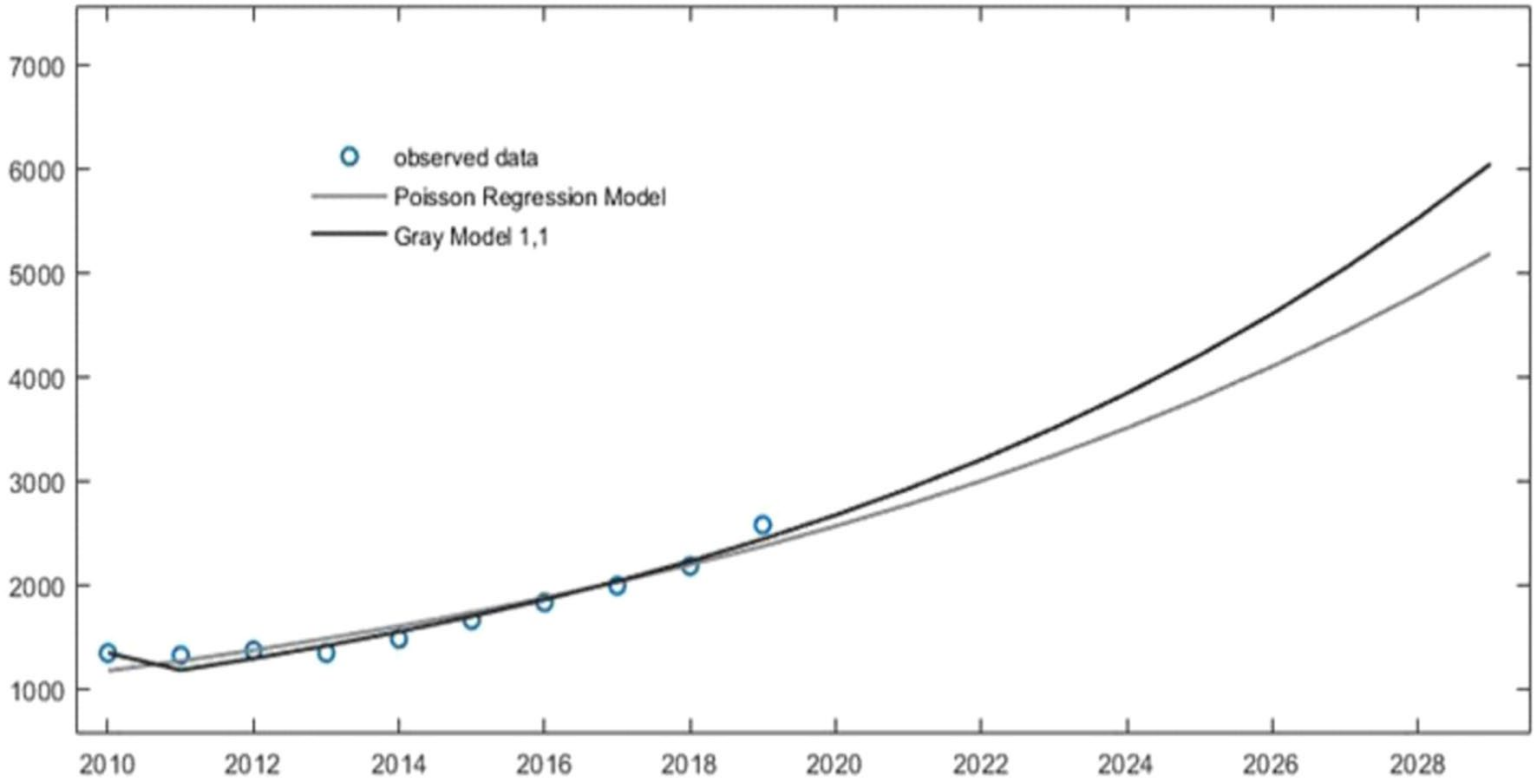
Projected modelling of hypertrophic cardiomyopathy to the next decades. *HCM* hypertrophic myocardiopathy.

Similar results were obtained using both Poisson regression analysis and GM (1,1) for the modelling prediction of new cases of HCM beyond 2019 based on the assumption that HCM incidence kept the same increasing trend. Figure 3 and Table 3 shows a 2.19-fold (Incidence rate in 2029:20.57, 95%CI: 20.44–21.56) increase in incident HCM cases by 2029 using Poisson regression analysis and a 2.48-fold (Incidence rate in 2019: 24.78, 95%CI: 24.38–25.62) increase in 2029 using GM (1,1).

**Table 3:** Modelling predicted number of hypertrophic myocardiopathy over the next decades.

Last decades	Year	2010	2011	2012	2013	2014	2015	2016	2017	2018	2019
Observed number		1343	1320	1368	1344	1479	1659	1829	1989	2179	2575
Predicted number	Poisson regression model	1171	1267	1370	1482	1602	1733	1874	2026	2191	2370
	Gray model (1,1)	1343	1179	1291	1414	1548	1695	1856	2033	2226	2438

Next decades	Year	2020	2021	2022	2023	2024	2025	2026	2027	2028	2029
Predicted number	Poisson regression model	2562	2771	2997	3241	3505	3790	4098	4432	4793	5183
	Gray model (1,1)	2669	2923	3201	3505	3839	4204	4603	5041	5520	6045

For Gray model (1,1) analysis, the *P* value is 1 and the C value is 0.19.

The modeling projections of the subtypes of incident HCM cases (including apical HCM, obstructive HCM, unobstructive HCM and undefined HCM) are shown in Supplementary Fig. III. and the estimated predicting numbers of incidence of subtypes of HCM cases are shown in Supplementary Table VI. This showed two to three-fold increase of incident HCM by 2029 compared to 2019.

### Incidence of mortality in HCM

Percentages of death are described in Supplementary Fig. IV. Of 17,085 HCM cases, 3.26% (*N* = 556) died during the studied time period. The overall mortality for HCM increased from 2.38% (*N* = 32) in 2010 to 3.38% (*N* = 87) in 2019. The mortality was stable in each year (*p* < 0.001) while the incidence of HF increased from 2010 to 2019 in overall, and amongst males and females (Table 2). No significant change could be seen for the mortality in the first (3.15%, *N* = 216) and last five years (3.32%, *N* = 340, *p* = 0.56). Males had a lower mortality (2.70% vs. 4.20%, *p* < 0.001) compared to females (Supplementary Table VII). One thousand and sixty-six (6.24%) of them experienced ventricular tachycardia or fibrillation from 2010 to 2019, keeping stable from 2010 to 2019 (*p* = 0.25). We also found that 1.40% (*N* = 239) of HCM patients received ICD over the past 10 years, and the implantation rate increased from 0.74% (*N* = 10) in 2010 to 1.32% (*N* = 34) in 2019, showing an overall upward trend (*p* = 0.25). In our study, the mortality of patients with ICD implantation was 3.35% (*N* = 8), while the mortality of patients without ICD implantation was 3.25% (*N* = 548), which did not show statistical significance (*p* = 0.85) (Supplementary Table VIII).

## Discussion

In this study, our principal findings are as follows: (i) The prevalence of HCM was 0.0069% in 2010 and rising to 0.076% in 2019, and the rising incidence of 6.85 per 1000 000 person-year in 2010 to 11.76 per 1000 000 person-year in 2019; (ii) the AAPC for the rising incidence of HCM was 5.8% and the expected number will double by 2029; (iii) HCM had stable mortality during the studied period; and (iv) males had a lower mortality compared to females.

Almost 20 years ago, the estimated prevalence of HCM was reported of 0.2%, which was based on the CARDIA (Coronary Artery Risk Development in Young Adults) cohort study originally collecting information of people aged 23 to 35 years using phenotype-based echocardiographic examinations^3^. Furthermore, HCM-causing pathogenic mutations involving sarcomere mutations suggested a prevalence of 0.5% or greater^9,20,21^. An earlier HCM screening study in patients age 18–74 years old found a prevalence of HCM of 0.16% in China^7^. We speculated that the differences of prevalence between our study and previous studies were mainly caused by the different studied populations, though our research methods were similar to some studies^7^. We used the data from the real-world administrative data, rather than screening specifically for HCM, and confirmed the detected HCM incidence remained low in the Chinese population, as the prevalence of HCM was 0.076% of our 21.90 million population covering all ages. Besides, our study involved all the centers around the whole Beijing district, the metropolis where the inhabitants come from all over the country. Furthermore, this study involved diverse racial residents in the area of Beijing.

The smaller prevalence of HCM than previous studies suggests that the diagnosis was much lower than expected. Data of BMHCIC provide robust evidence for the epidemiological status of clinically relevant HCM in the last decades. Unidentified HCM is likely to greater, since the diagnosis of HCM was largely dependent on the clinical manifestation and the willing to seek medication of the patients. We also found that the mortality in our study was higher than in previous studies (about 1% per year). Unfortunately, our high mortality is not related to ICD implantation.

This is the first study revealing a rising trend of HCM incidence in the Chinese population, similar to the rising prevalence noted in Korea over the last decades^6^. The incidence of HCM showed an increasing trend by AAPC from 2010 to 2019, and such increase trends were found both in males and females. However, this increase trend vanished when the analysis was restricted to the age range of 0–9 years and older than 60 years. The increased incidence risk of HCM with ageing was reported previously^22^. This phenomenon of rapid rising HCM incidence in the social active population of age ranging from 10 to 59 years old may provide some clues that the rising HCM mutation can be related to the rapid economy development, environmental and lifestyle changes in Asia in recent years.

By assuming the same increase trend over the next decades, we explored the possible future HCM number and incidence. The clinical diagnosis of HCM can perhaps be considered as “the tip of the iceberg of the disease spectrum” indicating an underestimation of the incidence of HCM^2^. Subtypes of HCM had broadly similar trends with ageing to the incidence of overall HCM. Their current status and modelling projections to the next decades were mostly consistent with the overall data for HCM.

### Limitations

There are some limitations to be mentioned for the current study. Like some studies investigating HCM prevalence, we used clinically diagnosed data mostly by echocardiographic examinations or CMR results and only a minority of the patients had genetic examination results. Second, because not all the admitted patients had echo-cardiography examinations, the lower prevalence than previous studies may indicated a lower identification rate of HCM in China. Therefore, raising attention to the HCM screening is necessary to reduce their complications, such as heart failure, stroke or even sudden cardiac death. Third, this study was not specifically designed as a screening study for HCM.

## Conclusions

The calculated HCM prevalence was much lower compared to prior screening studies from 2004, although the predicted HCM incidence would double over the next decades. HCM was associated with a stable mortality during the studied period.

## Supplementary Information

Supplementary Information.

## Data Availability

All data generated or analysed during this study are included in this published article and its supplementary information files.
