# Supplementary material for: Prevalence, incidence and mortality of hypertrophic cardiomyopathy based on a population cohort of 21.9 million in China
Source: Sci Rep. 2022 Nov 5;12:18799. doi: 10.1038/s41598-022-20042-9 (PMC9637201; doi:10.1038/s41598-022-20042-9)
Supplement: Supplementary file 1 — Supplementary Information. [file 41598_2022_20042_MOESM1_ESM.docx]

**Prevalence, incidence and mortality of hypertrophic Cardiomyopathy based on a population cohort of 21.9 million in China**

Ying Bai; Jian-Peng Zheng; Feng Lu; Xi-Lin Zhang; Chang-Ping Sun; Wei-Hua Guo; Yi-Xi Zou; Gregory YH Lip; Xu-Bo Shi

Supplementary Table I-VIII Page 2-15

Supplementary Figure I-V Page 16-20

| **Supplementary Table I.** Diagnostic indicators of hypertrophic cardiomyopathy | |
| --- | --- |
| Indicators | Values |
| Left Atrial Diameter, cm | 41.4±7.5 |
| Left Ventricular End-Diastolic Diameter, cm | 41.3±5.1 |
| LVEF, % | 62.5±3.3 |
| Myocardium Thickness, cm | 19.2±6.3 |
| Systolic mitral regurgitation bundle area, cm^2^ | 7.0±3.3 |
| LVOT-MV, cm/s | 302.4±233.7 |

| **Supplementary Table II** Prevalence of hypertrophic cardiomyopathy | | | |  |
| --- | --- | --- | --- | --- |
|  | HCM cases | Population | Prevalence (95% CI) | |
| Total |  |  |  | |
| 2010 | 1343 | 19612368 | 0.0069% (0.0065%, 0.0072%) | |
| 2011 | 2631 | 20231159 | 0.013% (0.013%, 0.014%) | |
| 2012 | 3966 | 20767977 | 0.019% (0.019%, 0.020%) | |
| 2013 | 5260 | 21246815 | 0.025% (0.024%, 0.025%) | |
| 2014 | 6690 | 21703661 | 0.031% (0.030%, 0.032%) | |
| 2015 | 8297 | 21875603 | 0.038% (0.037%, 0.039%) | |
| 2016 | 10065 | 21957104 | 0.046% (0.045%, 0.047%) | |
| 2017 | 11994 | 21947103 | 0.055% (0.054%, 0.056%) | |
| 2018 | 14106 | 21920099 | 0.064% (0.063%, 0.065%) | |
| 2019 | 16616 | 21904097 | 0.076% (0.075%, 0.077%) | |
| Males |  |  |  | |
| 2010 | 824 | 10126430 | 0.0081% (0.0076%, 0.0087%) | |
| 2011 | 1644 | 10318297 | 0.016% (0.015%, 0.017%) | |
| 2012 | 2504 | 10733216 | 0.023% (0.022%, 0.024%) | |
| 2013 | 3312 | 10975131 | 0.030% (0.029%, 0.031%) | |
| 2014 | 4204 | 11184057 | 0.038% (0.036%, 0.039%) | |
| 2015 | 5165 | 11258031 | 0.046% (0.045%, 0.047%) | |
| 2016 | 6342 | 11262398 | 0.056% (0.055%, 0.058%) | |
| 2017 | 7584 | 11240396 | 0.067% (0.066%, 0.069%) | |
| 2018 | 8945 | 11223394 | 0.080% (0.078%, 0.081%) | |
| 2019 | 10537 | 11206391 | 0.094% (0.092%, 0.096%) | |
| Females |  |  |  | |
| 2010 | 519 | 9483938 | 0.0055% (0.0050%, 0.0059%) | |
| 2011 | 987 | 9912861 | 0.010% (0.009%, 0.011%) | |
| 2012 | 1462 | 10034761 | 0.015% (0.014%, 0.015%) | |
| 2013 | 1948 | 10271684 | 0.019% (0.018%, 0.020%) | |
| 2014 | 2486 | 10519604 | 0.024% (0.023%, 0.025%) | |
| 2015 | 3132 | 10617573 | 0.030% (0.028%, 0.031%) | |
| 2016 | 3723 | 10694705 | 0.035% (0.034%, 0.036%) | |
| 2017 | 4410 | 10706706 | 0.041% (0.040%, 0.042%) | |
| 2018 | 5161 | 10696705 | 0.048% (0.047%, 0.050%) | |
| 2019 | 6079 | 10697705 | 0.057% (0.055%, 0.058%) | |

HCM, hypertrophic cardiomyopathy

**Supplementary Table III.** Annual incidence of Subtypes of HCM from 2010 to 2019

| Year | Total | | Male | | Female | |
| --- | --- | --- | --- | --- | --- | --- |
| Apical HCM | New cases | Incidence (95% CI) | New cases | Incidence (95% CI) | New cases | Incidence (95% CI) |
| 2010 | 90 | 0.46(0.36,0.55) | 62 | 0.61(0.46,0.76) | 28 | 0.3(0.19,0.4) |
| 2011 | 111 | 0.55(0.45,0.65) | 76 | 0.74(0.57,0.9) | 35 | 0.35(0.24,0.47) |
| 2012 | 107 | 0.52(0.42,0.61) | 77 | 0.72(0.56,0.88) | 30 | 0.3(0.19,0.41) |
| 2013 | 101 | 0.48(0.38,0.57) | 65 | 0.59(0.45,0.74) | 36 | 0.35(0.24,0.46) |
| 2014 | 115 | 0.53(0.43,0.63) | 80 | 0.72(0.56,0.87) | 35 | 0.33(0.22,0.44) |
| 2015 | 132 | 0.6(0.5,0.71) | 88 | 0.78(0.62,0.94) | 44 | 0.41(0.29,0.54) |
| 2016 | 166 | 0.76(0.64,0.87) | 125 | 1.11(0.92,1.3) | 41 | 0.38(0.27,0.5) |
| 2017 | 168 | 0.77(0.65,0.88) | 124 | 1.1(0.91,1.3) | 44 | 0.41(0.29,0.53) |
| 2018 | 196 | 0.89(0.77,1.02) | 129 | 1.15(0.95,1.35) | 67 | 0.63(0.48,0.78) |
| 2019 | 256 | 1.17(1.03,1.31) | 184 | 1.64(1.4,1.88) | 72 | 0.67(0.52,0.83) |
| Obstructive HCM |  | |  | |  | |
| 2010 | 436 | 2.22(2.01,2.43) | 236 | 2.33(2.03,2.63) | 200 | 2.11(1.82,2.4) |
| 2011 | 419 | 2.07(1.87,2.27) | 234 | 2.27(1.98,2.56) | 185 | 1.87(1.6,2.14) |
| 2012 | 438 | 2.11(1.91,2.31) | 254 | 2.37(2.08,2.66) | 184 | 1.83(1.57,2.1) |
| 2013 | 480 | 2.26(2.06,2.46) | 274 | 2.5(2.2,2.79) | 206 | 2.01(1.73,2.28) |
| 2014 | 534 | 2.46(2.25,2.67) | 286 | 2.56(2.26,2.85) | 248 | 2.36(2.06,2.65) |
| 2015 | 669 | 3.06(2.83,3.29) | 366 | 3.25(2.92,3.58) | 303 | 2.85(2.53,3.18) |
| 2016 | 642 | 2.92(2.7,3.15) | 366 | 3.25(2.92,3.58) | 276 | 2.58(2.28,2.89) |
| 2017 | 745 | 3.39(3.15,3.64) | 421 | 3.75(3.39,4.1) | 324 | 3.03(2.7,3.36) |
| 2018 | 777 | 3.54(3.3,3.79) | 450 | 4.01(3.64,4.38) | 327 | 3.06(2.73,3.39) |
| 2019 | 1013 | 4.62(4.34,4.91) | 574 | 5.12(4.7,5.54) | 439 | 4.1(3.72,4.49) |
| Unobstructive HCM |  | |  | |  | |
| 2010 | 245 | 1.25(1.09,1.41) | 161 | 1.59(1.34,1.84) | 84 | 0.89(0.7,1.08) |
| 2011 | 230 | 1.14(0.99,1.28) | 156 | 1.51(1.27,1.75) | 74 | 0.75(0.58,0.92) |
| 2012 | 218 | 1.05(0.91,1.19) | 137 | 1.28(1.06,1.49) | 81 | 0.81(0.63,0.98) |
| 2013 | 266 | 1.25(1.1,1.4) | 181 | 1.65(1.41,1.89) | 85 | 0.83(0.65,1) |
| 2014 | 258 | 1.19(1.04,1.33) | 173 | 1.55(1.32,1.78) | 85 | 0.81(0.64,0.98) |
| 2015 | 314 | 1.44(1.28,1.59) | 200 | 1.78(1.53,2.02) | 114 | 1.07(0.88,1.27) |
| 2016 | 366 | 1.67(1.5,1.84) | 254 | 2.26(1.98,2.53) | 112 | 1.05(0.85,1.24) |
| 2017 | 383 | 1.75(1.57,1.92) | 261 | 2.32(2.04,2.6) | 122 | 1.14(0.94,1.34) |
| 2018 | 447 | 2.04(1.85,2.23) | 315 | 2.81(2.5,3.12) | 132 | 1.23(1.02,1.44) |
| 2019 | 428 | 1.95(1.77,2.14) | 300 | 2.68(2.37,2.98) | 128 | 1.2(0.99,1.4) |
| Undefined HCM |  | |  | |  | |
| 2010 | 572 | 2.92(2.68,3.16) | 365 | 3.6(3.23,3.97) | 207 | 2.18(1.89,2.48) |
| 2011 | 560 | 2.77(2.54,3) | 375 | 3.63(3.27,4) | 185 | 1.87(1.6,2.14) |
| 2012 | 605 | 2.91(2.68,3.15) | 407 | 3.79(3.42,4.16) | 198 | 1.97(1.7,2.25) |
| 2013 | 497 | 2.34(2.13,2.54) | 313 | 2.85(2.54,3.17) | 184 | 1.79(1.53,2.05) |
| 2014 | 572 | 2.64(2.42,2.85) | 373 | 3.34(3,3.67) | 199 | 1.89(1.63,2.15) |
| 2015 | 544 | 2.49(2.28,2.7) | 340 | 3.02(2.7,3.34) | 204 | 1.92(1.66,2.19) |
| 2016 | 655 | 2.98(2.75,3.21) | 458 | 4.07(3.69,4.44) | 197 | 1.84(1.58,2.1) |
| 2017 | 693 | 3.16(2.92,3.39) | 467 | 4.15(3.78,4.53) | 226 | 2.11(1.84,2.39) |
| 2018 | 759 | 3.46(3.22,3.71) | 502 | 4.47(4.08,4.86) | 257 | 2.4(2.11,2.7) |
| 2019 | 878 | 4.01(3.74,4.27) | 571 | 5.1(4.68,5.51) | 307 | 2.87(2.55,3.19) |

HCM, hypertrophic cardiomyopathy.

| **Supplementary Table IV.** IRR of male to female after adjustment of age | |
| --- | --- |
| HCM types | IRR (95% CI) |
| All HCM | 1.73(1.68,1.79) |
| Apical HCM | 2.4(2.69,2.14) |
| Obstructive HCM | 1.29(1.36,1.23) |
| Unobstructive HCM | 2.11(2.28,1.96) |
| Undefined HCM | 1.98(2.08,1.88) |

HCM, hypertrophic cardiomyopathy; IRR, incidence rate ratio.

| **Supplementary Table V**. Increasing trends of the subtypes of HCM | | | | | | |
| --- | --- | --- | --- | --- | --- | --- |
| Apical HCM | Year Range | Joinpoint | APC（95% CI） | p value | AAPC（95% CI） | p value |
| Total | 2010-2014 | 1 | 1.2（-10.0-13.9） | 0.8 |  |  |
|  | 2014-2019 | 1 | 16.5（9.6-23.8） | <0.05 |  |  |
|  | 2010-2019 | 1 |  |  | 9.4(4.4-14.8） | p<0.05 |
|  | 2010-2019 | 0 |  |  | 10.6 (7.2-14.2) | p<0.05 |
| Male | 2010-2013 | 1 | -2.8(-24.2-24.6) | >0.05 |  |  |
|  | 2013-2019 | 1 | 15.9(8.7-23.6) | <0.05 |  |  |
|  | 2010-2019 | 1 |  |  | 9.3(1.8-17.3) | p<0.05 |
|  | 2010-2019 | 0 |  |  | 11.2(7.0-15.5) | p<0.05 |
| Female | 2010-2017 | 1 | 5.3(0.8-10.0) | <0.05 |  |  |
|  | 2017-2019 | 1 | 28.9(2.3-62.4) | <0.05 |  |  |
|  | 2010-2019 | 1 |  |  | 10.1(5.1-15.4) | p<0.05 |
|  | 2010-2019 | 0 |  |  | 9.5(5.6-13.7) | p<0.05 |
| Obstructive HCM | Year Range | Joinpoint | APC（95% CI） | p value | AAPC（95% CI） | p value |
| Total | 2010-2012 | 1 | -3.8（-32.0-36.2） | 0.79 |  |  |
|  | 2012-2019 | 1 | 11.3（7.3-15.4） | <0.05 |  |  |
|  | 2010-2019 | 1 |  |  | 7.7(1.2-14.7） | p<0.05 |
|  | 2010-2019 | 0 |  |  | 9.2 (6.6-11.9) | p<0.05 |
| Male | 2010-2014 | 1 | 3.8(-4.9-13.3) | 0.33 |  |  |
|  | 2014-2019 | 1 | 13.4(8.2-18.8) | <0.05 |  |  |
|  | 2010-2019 | 1 |  |  | 9.0(5.2-13.0) | p<0.05 |
|  | 2010-2019 | 0 |  |  | 9.7(7.3-12.2) | p<0.05 |
| Female | 2010-2012 | 1 | -5.2(-38.7-46.5) | 0.76 |  |  |
|  | 2012-2019 | 1 | 10.8(5.8-16.1) | <0.05 |  |  |
|  | 2010-2019 | 1 |  |  | 7.0(-1.1-15.8) | p>0.05 |
|  | 2010-2019 | 0 |  |  | 8.6(5.6-11.7) | p<0.05 |
| Unobstructive HCM | Year Range | Joinpoint | APC（95% CI） | p value | AAPC（95% CI） | p value |
| Total | 2010-2012 | 1 | -6.7（-31.0-26.0） | 0.58 |  |  |
|  | 2012-2019 | 1 | 10.0（6.4-13.8） | <0.05 |  |  |
|  | 2010-2019 | 1 |  |  | 6.1(0.4-12.0） | p<0.05 |
|  | 2010-2019 | 0 |  |  | 7.6 (4.9-10.3) | p<0.05 |
| Male | 2010-2012 | 1 | -8.0(-36.4-33.1) | 0.59 |  |  |
|  | 2012-2019 | 1 | 11.4(6.9-16.1) | <0.05 |  |  |
|  | 2010-2019 | 1 |  |  | 6.8(-0.2-14.2) | p>0.05 |
|  | 2010-2019 | 0 |  |  | 8.6(5.4-11.8) | p<0.05 |
| Female | 2010-2012 | 1 | -4.1(-30.5-32.4) | 0.75 |  |  |
|  | 2012-2019 | 1 | 7.3(3.3-11.3) | <0.05 |  |  |
|  | 2010-2019 | 1 |  |  | 4.6(-1.3-11.0) | p>0.05 |
|  | 2010-2019 | 0 |  |  | 5.6(3.3-8.0) | p<0.05 |
| Undefined HCM | Year Range | Joinpoint | APC（95% CI） | p value | AAPC（95% CI） | p value |
| Total | 2010-2015 | 1 | %-2.7（-7.7-2.6） | 0.24 |  |  |
|  | 2015-2019 | 1 | 12.2（5.1-19.7） | <0.05 |  |  |
|  | 2010-2019 | 1 |  |  | 3.6(0.4-7.0） | p<0.05 |
|  | 2010-2019 | 0 |  |  | 3.8 (0.6-7.0) | p<0.05 |
| Male | 2010-2015 | 1 | %-2.4(-9.0-4.7) | 0.41 |  |  |
|  | 2015-2019 | 1 | 12.4(3.3-22.2) | <0.05 |  |  |
|  | 2010-2019 | 1 |  |  | 3.9(-0.3-8.3) | p>0.05 |
|  | 2010-2019 | 0 |  |  | 4.0(0.5-7.6) | p<0.05 |
| Female | 2010-2012 | 1 | %-1.9(-5.2-1.6) | 0.22 |  |  |
|  | 2012-2019 | 1 | 16.1(6.2-26.9) | <0.05 |  |  |
|  | 2010-2019 | 1 |  |  | 3.8(0.8-6.8) | p>0.05 |
|  | 2010-2019 | 0 |  |  | 3.3(0.1-6.7) | p<0.05 |
|  |  |  |  |  |  |  |

| **Supplementary Table VI.** Modelling Predicted Number of subtypes of HCM to the next Decades | | | | | | | | | | | |
| --- | --- | --- | --- | --- | --- | --- | --- | --- | --- | --- | --- |
| **Apical HCM** | | | | | | | | | | | |
| Lase Decades | Year | 2010 | 2011 | 2012 | 2013 | 2014 | 2015 | 2016 | 2017 | 2018 | 2019 |
| Observed Number | | 90 | 111 | 107 | 101 | 115 | 132 | 166 | 168 | 196 | 256 |
| Predicted Number | PS | 90 | 85 | 97 | 109 | 124 | 141 | 159 | 181 | 205 | 232 |
|  | GM | 83 | 93 | 104 | 116 | 130 | 145 | 162 | 181 | 203 | 227 |
| Next Decades | Year | 2020 | 2021 | 2022 | 2023 | 2024 | 2025 | 2026 | 2027 | 2028 | 2029 |
| Predicted Number | PS | 263 | 298 | 337 | 382 | 433 | 491 | 556 | 631 | 715 | 810 |
|  | GM | 253 | 283 | 317 | 354 | 396 | 443 | 496 | 554 | 620 | 693 |
| **Obstructive HCM** | | | | | | | | | | | |
| Lase Decades | Year | 2010 | 2011 | 2012 | 2013 | 2014 | 2015 | 2016 | 2017 | 2018 | 2019 |
| Observed Number | | 436 | 419 | 438 | 480 | 534 | 669 | 642 | 745 | 777 | 1013 |
| Predicted Number | PS | 436 | 392 | 438 | 488 | 545 | 608 | 678 | 757 | 844 | 942 |
|  | GM | 379 | 419 | 462 | 510 | 563 | 621 | 686 | 757 | 835 | 922 |
| Next Decades | Year | 2020 | 2021 | 2022 | 2023 | 2024 | 2025 | 2026 | 2027 | 2028 | 2029 |
| Predicted Number | PS | 1051 | 1173 | 1309 | 1460 | 1629 | 1817 | 2028 | 2262 | 2524 | 2816 |
|  | GM | 1017 | 1123 | 1239 | 1368 | 1509 | 1666 | 1839 | 2029 | 2240 | 2472 |
| **Unobstructive HCM** | | | | | | | | | | | |
| Lase Decades | Year | 2010 | 2011 | 2012 | 2013 | 2014 | 2015 | 2016 | 2017 | 2018 | 2019 |
| Observed Number | | 245 | 230 | 218 | 266 | 258 | 314 | 366 | 383 | 447 | 428 |
| Predicted Number | PS | 245 | 215 | 237 | 260 | 286 | 314 | 345 | 379 | 416 | 457 |
|  | GM | 209 | 228 | 248 | 270 | 294 | 320 | 348 | 378 | 412 | 448 |
| Next Decades | Year | 2020 | 2021 | 2022 | 2023 | 2024 | 2025 | 2026 | 2027 | 2028 | 2029 |
| Predicted Number | PS | 503 | 552 | 607 | 667 | 732 | 805 | 884 | 971 | 1067 | 1173 |
|  | GM | 488 | 531 | 577 | 628 | 684 | 744 | 810 | 881 | 959 | 1043 |
| **Undefined HCM** | | | | | | | | | | | |
| Lase Decades | Year | 2010 | 2011 | 2012 | 2013 | 2014 | 2015 | 2016 | 2017 | 2018 | 2019 |
| Observed Number | | 572 | 560 | 605 | 497 | 572 | 544 | 655 | 693 | 759 | 878 |
| Predicted Number | PS | 572 | 493 | 524 | 557 | 593 | 631 | 671 | 713 | 759 | 807 |
|  | GM | 505 | 530 | 556 | 584 | 612 | 643 | 675 | 708 | 743 | 780 |
| Next Decades | Year | 2020 | 2021 | 2022 | 2023 | 2024 | 2025 | 2026 | 2027 | 2028 | 2029 |
| Predicted Number | PS | 859 | 913 | 971 | 1033 | 1099 | 1169 | 1243 | 1322 | 1406 | 1496 |
|  | GM | 819 | 859 | 902 | 946 | 993 | 1042 | 1094 | 1148 | 1205 | 1265 |

P value and C value for Apical HCM was 1 and 0.27, for obstructive HCM of 1.00 and 0.22, for unobstructive HCM of 1.00 and 0.24, and for undefined HCM of 0.8 and 0.49, respectively.

| **Supplementary Table VII.** Comparison of HCM mortality between 5 year range and sex | | | | | |
| --- | --- | --- | --- | --- | --- |
| Year range comparison | | Event | HCM Number | Crude rate | p value |
| death | 2010-2014 | 216 | 6854 | 0.03151444 | 0.56 |
|  | 2015-2019 | 340 | 10231 | 0.03323233 |  |
| Men vs. Women | |  |  |  |  |
| death | Men | 291 | 10780 | 0.02699443 | <0.001 |
|  | Women | 265 | 6305 | 0.04203013 |  |

HF, heart failure; HCM, hypertrophic cardiomyopathy

| **Supplementary Table VIII.** Mortality in hypertrophic cardiomyopathy | | | | |
| --- | --- | --- | --- | --- |
|  | Newly diagnosed HCM cases | Annual number of deaths | Number of ICD implants | Number of deaths after ICD implantation |
| Total | 17085 | 556 | 239 | 8 |
| 2010 | 1343 | 32(2.38%) | 10 (0.74%) | 0 |
| 2011 | 1320 | 33(2.50%) | 11 (0.83%) | 0 |
| 2012 | 1368 | 50(3.65%) | 23 (1.68%) | 0 |
| 2013 | 1344 | 49(3.65%) | 19 (1.41%) | 1 (5.26%) |
| 2014 | 1479 | 52(3.52%) | 23 (1.56%) | 1 (4.35%) |
| 2015 | 1659 | 61(3.68%) | 24 (1.45%) | 0 |
| 2016 | 1829 | 60(3.28%) | 27 (1.48%) | 2 (7.41%) |
| 2017 | 1989 | 67(3.37%) | 27 (1.36%) | 2 (7.41%) |
| 2018 | 2179 | 65(2.98%) | 41 (1.88%) | 1 (1.72%) |
| 2019 | 2575 | 87(3.38%) | 34 (1.32%) | 1 (2.94%) |

HCM, hypertrophic cardiomyopathy; Implantable Cardioverter-defibrillator

A.


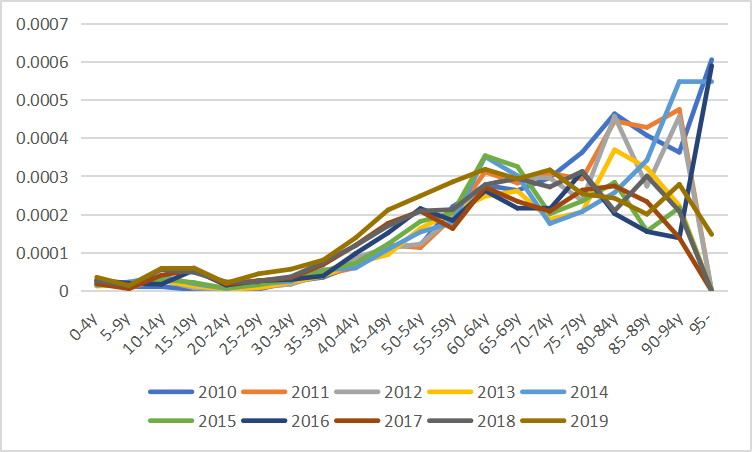


B.

**
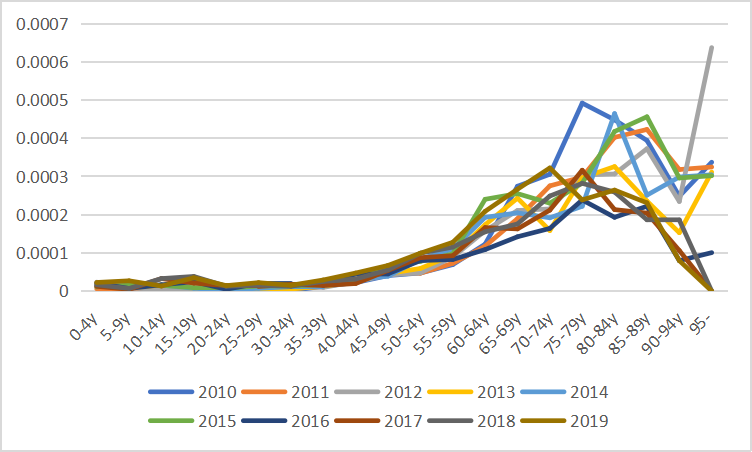
**

**Supplementary Figure I. The Age-specific incidence rate curves of HCM in males and Females.**


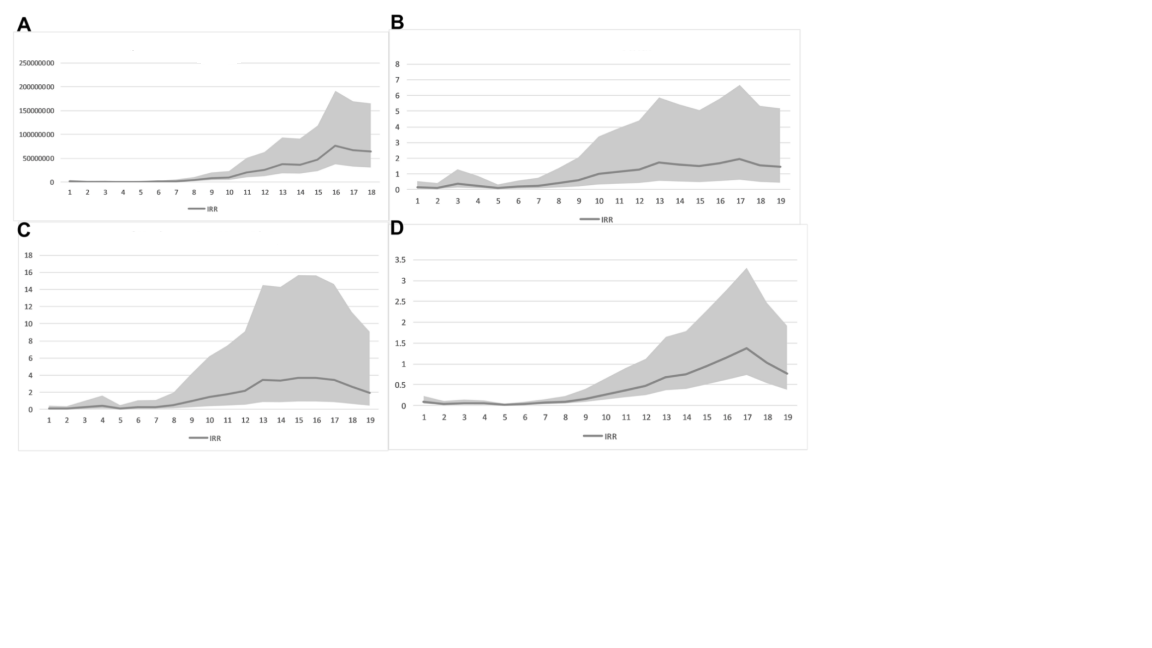


**Supplementary Figure II.** Incidence of subtypes of HCM (A. Apical HCM; B. Obstructive HCM; C. Unobstructive HCM; D. Undefined HCM) with Ageing. Gray Shadow represents 95% confidence interval of corresponding IRR; HCM, hypertrophic myocardiopathy. IRR, incidence rate ratio.

Age range:1,0-4 years; 2, 5-9 years; 3,10-14 years; 4, 15-19 years; 5, 20-24 years; 6, 25-29 years; 7,30-34 years; 8,35-39 years;9,40-44 years; 9, 40-44 years; 10,45- 49 years; 11,50-54 years; 12, 55-59 years; 13, 60-64 years; 14, 65-69 years; 15,70-74 years;16,75-79 years; 17,80-84 years; 18,85-89 years; 19, 90-94 years; 20 ,95+ years.

**
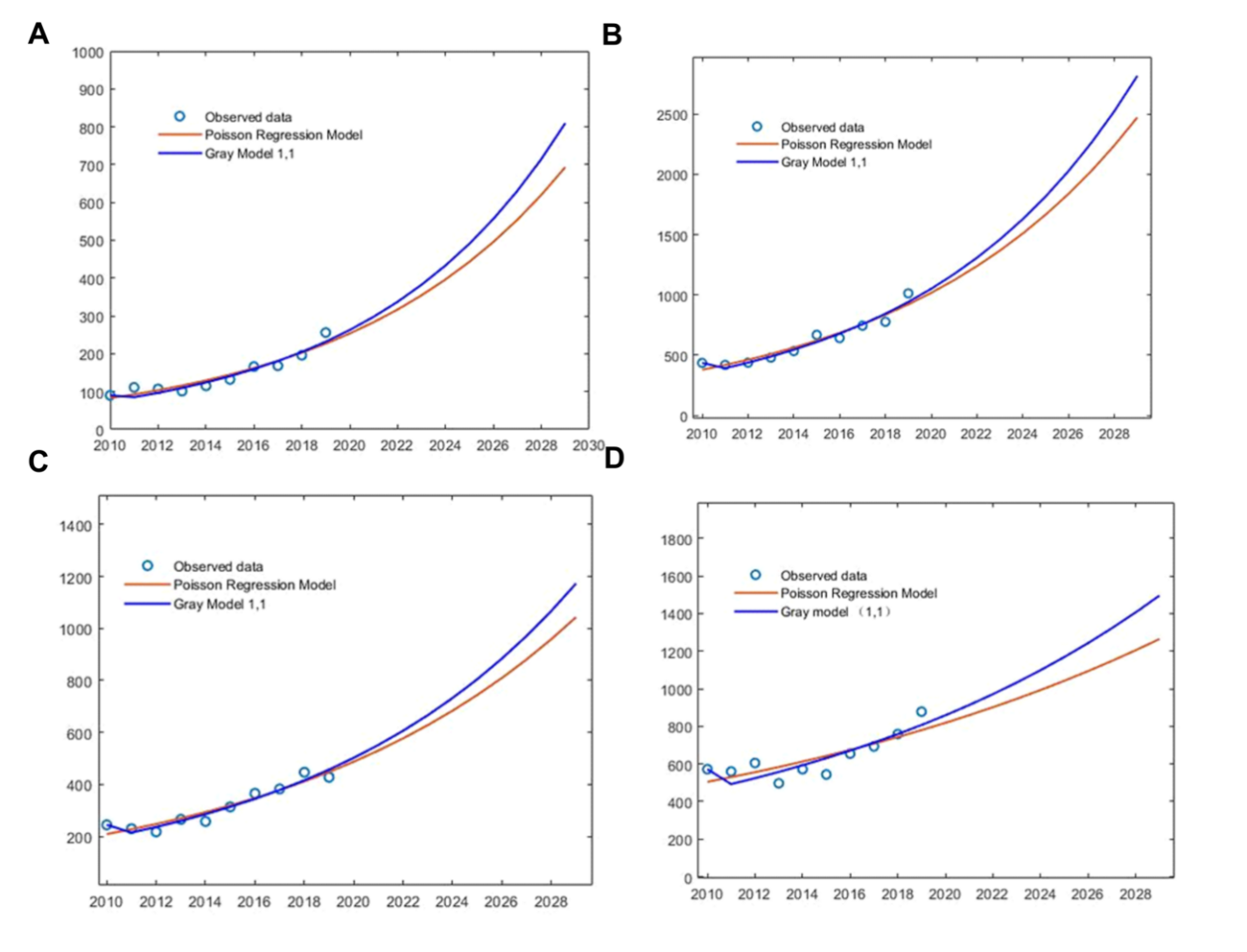
**

**Supplementary Figure III.** Projected modelling to the next decades of subtypes of HCM. (A. Apical HCM; B. Obstructive HCM; C. Unobstructive HCM; D. Undefined HCM). HCM, hypertrophic cardiomyopathy.

**Supplementary Figure IV.** Incidence of mortality in HCM with year


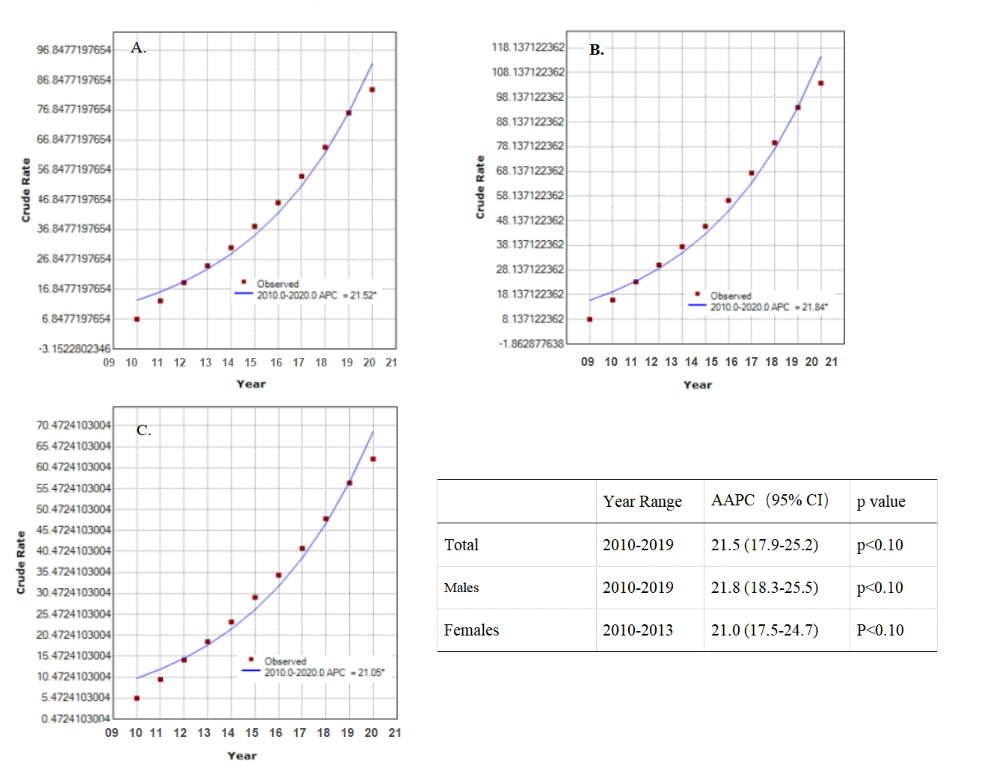


**Supplementary Figure V.** AAPC of the prevalence of HCM (A. Total population; B. Males; C. Females).
